# The course of radiographic loosening, pain and functional outcome around the first revision of a total hip arthroplasty

**DOI:** 10.1186/1471-2474-14-167

**Published:** 2013-05-15

**Authors:** Emin Aghayev, Regula Teuscher, Michal Neukamp, Eu Jin Lee, Markus Melloh, Stefan Eggli, Christoph Röder

**Affiliations:** 1 Institute for Evaluative Research in Orthopedic Surgery, University of Bern, Stauffacherstrasse 78, CH-3014, Bern, Switzerland; 2 Sonnenhof Clinic, Buchserstrasse 30, CH-3006, Bern, Switzerland; 3 Western Australian Institute for Medical Research, University of Western Australia, B Block, Hospital Avenue, Nedlands WA 6009, Australia

**Keywords:** Total hip arthroplasty, Revision THA, IDES, Radiographic loosening, Functional outcome

## Abstract

**Background:**

The published data on pain and physical function before and after revision of total hip arthroplasty (THA) is scarce. The study reports the course and interrelationships of radiographic loosening, pain and physical function 5 year before and after a first revision THA.

**Methods:**

The study was based on the IDES-THA database. All patients with their first THA revision for aseptic loosening and a documented index surgery on the same side and at least one pre-revision and one post-revision follow-up were selected. Only patients with an intact contralateral hip joint (Charnley class-A) were included. Follow-ups within ±5.5 years around the revision time point were analyzed. Annual prevalences of radiographic component loosening and the non-desired outcomes (moderate/severe/intolerable pain, walking <30 minutes, hip flexion range <90°) were calculated.

**Results:**

Signs of radiographic component loosening started to increase about 4 years before revision surgery. Two years later, a sharp increase of painful hips from 15% to 80% in the revision year was observed. In the year after revision surgery, this rate dropped back to below 10%. Walking capacity started to noticeably deteriorate 3 years before revision and in the revision year about 65% of patients could not walk longer than 30 minutes. As opposed to pain, walking capacity did not recover to pre-revision levels and the best outcome was only reached two years post-revision. Hip flexion range had the slowest and least extent of deterioration (≈45% flexed <70° in the revision year) but with the best outcomes at only three years after revision surgery it took the longest to recover.

**Conclusion:**

Prevalence of radiological loosening signs and/or pain intensity follow an almost parallel course around the first revision of a THA for aseptic component loosening. This process begins about 4 years (radiographic loosening) before the actual revision surgery and intensifies about 2 years later (pain). It also involves walking capacity and hip range of motion. While pain levels go back to levels similar to those after primary surgery, range of motion and even more walking capacity remain moderately compromised.

## Background

Total hip arthroplasty (THA) represents a considerable part of day-to-day orthopaedic routine and revision surgery is becoming more and more frequent and relevant. THA represents a key treatment for re-establishing independence and quality of life both in younger and in elderly patients with hip diseases. The main reason for revision surgery in THA is aseptic fixation failure. Using Ontario 1984–1994 discharge data, Coyte et al. derived an annual growth rate of 5.1% per year in the number of THA revisions [1]. Kurtz et al. formulated projections for the number of primary and revision total hip and knee arthroplasties that will be performed in the United States through 2030. He described that the growing demand of hip revision procedures will double the 2005 procedure numbers by the year 2026 and will increase to 96’700 annual procedures in 2030 [2].

Because of this development and because of highly demanding patient claims for mobility and quality of life even after revision surgery the course of implant component loosening, hip pain and function before and after revision total hip arthroplasty (RTHA) needs to be assessed. Survival analyses in the literature show revision rates around 5.7-5.9% for an average follow-up time of 7.4, 6 and 4.8 years, respectively [3-5]. Several studies on functional outcome after RTHA surgery have already been published [3,4,6]. In a meta-analysis Saleh et al. outlined that 67% of patients experience good to excellent Harris Hip Scores after revision surgery. His analysis confirmed that RTHA is generally less successful regarding functional outcome, morbidity and mortality rates than primary THA. Also, the study by Espehaug et al. showed that the pain alleviation and improvement of walking ability and need of help after THA was considerably poorer among patients who underwent revision surgery than among patients with primary surgery only [6].

In 1965 M.E. Müller started a systematic collection of THA outcome data and developed a documentation system that culminated in the International Documentation and Evaluation System (IDES) for total hip and knee arthroplasty [7,8]. IDES and precursors have collected prospective information about 48’000 primary THA, 12’000 RTHA, and 77’000 follow-ups from 65 hospitals in Europe.

Based on the comprehensive and detailed data pool, the current study aimed at description of pre- and postoperative pain and functional status of patients undergoing a first revision of THA and its relation with signs of radiographic component loosening.

## Methods

The current study is based on the IDES hip registry of the Institute for Evaluative Research in Orthopedic Surgery at the University of Bern. The history and administration of the registry have been previously described [7,8]. Institutional review board approval for the study was not required as it utilized existing anonymous observational data.

### Definition of cases

Assessment of component status was performed based on standardized antero-posterior pelvic and lateral radiographs with the MEM-template for the evaluation of THA as a standardized measurement tool [9]. Acetabular and femoral loosening was defined by comparing the postoperative and follow-up radiographs and measuring superior and medial migration and the tilt of the cup [10,11], radiolucencies around it [12], a broken cup [13] or broken cement [11], subsidence of the stem [14], radiolucencies at the stem-bone or cement-bone interface [13-15], a progressive tilt of the stem and cavitation and fracture of the stem [11].

Table 1 shows the clinical and radiographic variables used for describing pain, functional outcome and radiographic loosening [16].

**Table 1:** International documentation and evaluation system variables used for the study

**A (surgery form)**	**B (revision form)**	**C (follow-up form)**
Surgery date	Revision date	Follow-up date
Gender	Diagnosis	Hip pain degree
Birth date	N of previous revisions	Time walked without support
	Status of co-lateral hip	Hip flexion range
	Acetabular superior migration*	Acetabular superior migration*
	Acetabular medial migration*	Acetabular medial migration*
	Brocken implant*	Continuous radiolucency around cup*
	Stem subsidence*	Radiolucency between stem and cement*
	Stem out of shaft*	Radiolucency between bone and cement*
	Endosteal resorption*	Stem subsidence*
		Progressive tilt of stem*
		Endosteal resorption (small cavities only, defects)*
		Fracture of cement (femur, stem)*

Asterisk marks questions which defined signs of radiological loosening.

### Sample selection

All patients with their first THA revision and a documented index surgery on the same side and at least one pre-revision and one post-revision follow-up were selected. The linkage between follow-up and revision forms was carried out on the joint level. Only patients with an intact contralateral hip joint (Charnley class-A) were included.

A further inclusion criterion was a revision diagnosis of aseptic loosening. Patients with infection, fracture or other revision diagnoses (girdlestone, malposition, dislocation, trochanter pathology, etc.) were excluded. Patients with a second or multiple revisions were also excluded. Replacement of at least one component of the hip arthroplasty during revision surgery (acetabular only, femoral only, both) was mandatory. Only revisions later than five years after primary surgery were considered for the analysis in order to exclude early aseptic loosening. Only follow-ups within ±5.5 years around the revision time point were analyzed.

Applying these selection criteria 234 revised patients were found. Average interval between primary and revision surgery was 8.4 years (range 5–23 years). The patient sample had 543 pre-revision (average 2.3 per patient) and 410 post-revision (average 1.8 per patient) follow-ups.

There were 52.2% female patients. Average age at the time of surgery was 60.9 years (range 26.4-80.7 years) and average BMI was 26.8 kg/m^2^ (range 19–40). Average age at the revision was 69.1 years (range 36.1-88.0 years).

### Outcomes

The main outcome parameters were defined according to Bryant et al. [17]. He identified three core factors for analyzing the functional outcome after THA by comparing 13 methods of scoring THA outcomes. These three factors were pain, walking capacity and flexion range. Pain was classified as none/mild, moderate, or severe/intolerable; walking capacity was classified as more than 60 minutes, 31–60 minutes, 10–30 minutes, or less than 10 minutes/not possible; the range of hip flexion was classified as >90°, 71°-90°, 30°-70°, or <30°/stiff. We defined a poor outcome as moderate or severe/intolerable hip pain, a walking capacity of less than 30 minutes, and a range of hip flexion of ≤90°.

### Statistical analysis

All follow-up examinations were grouped on the basis of annual intervals for assessing the course of prevalences of undesired outcomes and radiographic component loosening. Relationships between pre- to post-revision clinical status (pain, walking, hip flexion range) were assessed performed using Spearman correlation.

The level of significance was set to 0.05 throughout the study. All statistical analyses were conducted using SAS 9.3 (SAS Institute Inc., Cary, NC, USA).

## Results

### Hip pain

**Figure 1:**
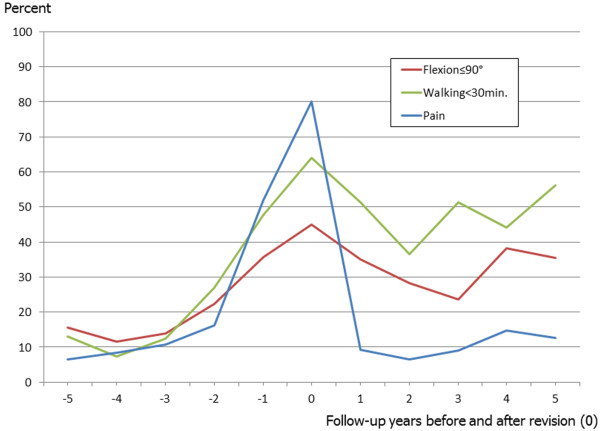
Proportions of patients with pain (moderate/severe/intolerable) and poor functional outcome (flexion ≤90° and walking<30 min.) over the course of the revision.

Figure 1 shows the consistently growing number of patients with relevant hip pain before revision from 6.5% five years before the revision up to 80% at the time of revision. After the revision, however, there is a strong decline to an average proportion of 10% during the following five years.

### Flexion range

As visible in Figure 1 the proportion of patients with hip flexion range ≤90° increases towards 45% in the revision year. After revision this proportion decreases again, but does not reach similarly low values than after the index surgery.

### Walking capacity

Similar to the decrease in hip flexion range and pain, the proportion of patients with a walking capacity <30 min. constantly increases up to 64% in the year of the revision (Figure 1). After revision surgery this proportion decreases again but it doesn’t either reach values of the post-primary phase.

### Signs of radiological loosening

**Figure 2:**
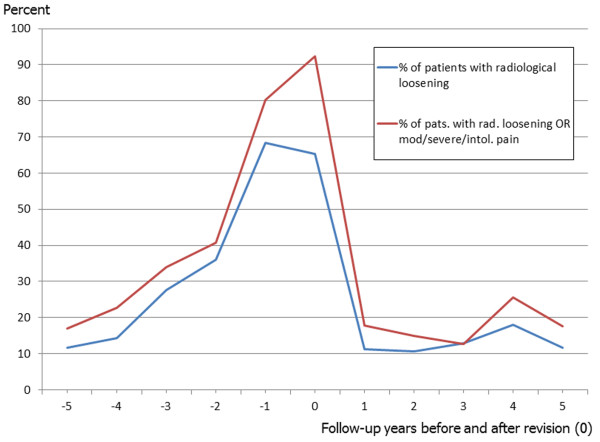
Proportions of patients with signs of radiological loosening before and after revision OR moderate/severe/intolerable pain.

As shown in Figure 2, the proportion of patients with signs of radiological loosening is constantly growing during the five years before revision. After revision there is a strong decline to post-primary values. The shape of the curve almost resembles that of the pain aggravation before and of pain alleviation after the revision.

### Correlation between pre- and post-revision patient clinical status

Analysis of relationship of pre- to post-revision patient clinical status showed small correlations between hip pain measured at the time of revision and at the first available post-revision follow-up (average follow-up time 1.46 years) (r=0.21; p=0.002) but moderate correlation of hip flexion (r=0.45; p<0.001) and of walking capacity (r=0.43; p<0.001).

If the clinical status at the time of revision was compared to the last available post-revision outcome (average follow-up time 2.74 years) the correlation for hip pain (r=0.25; p<0.001), hip flexion (r=0.52; p<0.001) and walking capacity (r=0.44; p<0.001) slightly increased.

## Discussion

### Summary of results

The analysis showed that the proportions of patients with radiological loosening signs are constantly increasing during the 4–5 years before an exchange of one or both mechanically loose components and that nearly all patients have a radiographically loose component and/or relevant, i.e. moderate, severe or intolerable pain in the revision year. The proportion of patients with a painful hip, restricted flexion and walking capacity continuously increases towards the revision year after which clear (hip pain), moderate (flexion range) or fair (walking capacity) improvement was seen. However, poor flexion was defined as flexion below 90° and poor walking capacity as one <30 minutes, which both may still be an acceptable result of a revision surgery in some patients.

### Course of pain relief

Pain relief and improved walking capacity are the probably two most important expectations of patients before primary hip arthroplasty [18]. This situation remains unchanged before a revision operation. Eisler reports that 92% of patients expect to have much less pain after surgery but at follow-up only 69% found their expectation fulfilled to a large or very large extent [19]. In the current investigation pain relief showed to be pleasingly strong and instantaneous after the first revision arthroplasty, and an average of about 90% of patients had no or only mild pain in the first five years post-revision, even if there was a slight deterioration of pain status starting around two years after surgery. In the study by Raut et al. 72.1% of patients were pain-free at a mean follow-up of 6 years after revision and further 21.4% had mild or occasional discomfort [4]. Though not stated, it can be assumed that other 6.5% had more severe pain. In our documented sample 12.5% of patient suffered from hip pain in the fifth post-revision year. Correlation of pain pre- to post-revision was small which supports the assumption that pain levels after the first revision total hip arthroplasty are generally low and independent of the pre-revision pain status. We already revealed a similar situation for the primary scenario [20].

### Course of functional outcome

Eisler further stated that 82% of patients expected similar walking capacities than after the primary operation or at least much improved ones, but only 55% found their expectations fulfilled to a large or very large extent [19]. He attributes walking capacity improvement as the probably most important reason for satisfaction or dissatisfaction after revision surgery. Similar to our previous findings after primary THA [20,21] where the best walking capacities were only achieved 2–3 years after surgery, the recovery of ambulation does also seem to be a process of at least 2 years. This may in part explain Eisler’s findings who measured outcome already at 1 year after revision surgery. In addition, our findings about the influence of pre-primary walking capacity on postoperative walking [19] and the correlation between pre-revision walking capacity and post-revision walking underlines the fact that patients’ expectations should be modest with regards to the potential for walking capacity improvement after the first revision of a THA. In the current study, an average of about 48% of patients had a walking capacity <30 minutes in the first five post-revision years.

Kershaw et al. [22] examined 159 patients at an average of about 6 years after revision THA and found 70% of hips to flex over 70°. These findings, although not directly comparable, seem to correspond well with the 65% of cases flexing over 90° in our study at five years after revision. The course of recovery of hip range of motion in the sagittal plane is the most delayed one in our study. It takes three years until the majority of patients reach a hip flexion range of 90° or beyond. Comparing hip flexion range with the preoperative levels we observed the best correlations, i.e. patients with good pre-revision hip flexion can also expect good motion after revision surgery, although its full recovery may take several years.

### Signs of radiographic loosening before and after revision

There is consensus that the detection and revision of loose THA components should happen early in order to preserve as much bone stock as possible [23,24]. The problem of asymptomatic destruction of bone stock by a loose component [25-28] makes regular radiographic follow-up the only viable monitoring option. Our previous research showed that chances for radiographic component loosening of asymptomatic or mildly symptomatic primary THAs are very low in the first five years after surgery but that they are increasing thereafter [16]. These findings are in line with Figure 2 where the prevalence of signs of radiographic loosening sharply increases about 4 years before the actual revision, which is about 4 years after the index surgery (average revision interval 8 years after index surgery), and with Figure 1 where the prevalence of moderate, severe or intolerable pain sharply increases about 2 years before the revision surgery, i.e. 6 years after the index surgery and with delay if compared with radiographic loosening signs. One needs to additionally consider Strömberg’s observations that there was an increased risk of loosening in hips where radiolucencies appeared within the first postoperative year, whereas an unchanged radiographic appearance after 1 year strongly indicated that the risk of later loosening was small. Consequently, asymptomatic hips with unchanged radiographic appearance after 1 year didn’t need to be radiographically monitored until year 5 after surgery whereas painful hips and those with early radiolucencies needed regular follow-up early on [29]. Surveying the Charnley Elite Plus hip system Ollivere found a similar relationship for later follow-up intervals in that radiological loosening at 6.4 years was predictive of failure at 12 years and concluding that medium-term radiographs and clinical scores should be included in the surveillance of THA to give an early indication of the performance of specific implants [30].

The survival of the revised implants until re-revision was not focus of the current study, but the prevalence of radiographic loosening signs ranged between 5-10% for cups and stems depending on follow-up year and remained relatively stable during the first five post-revision years. These findings correspond well with those of Izquierdo and Northmore-Ball who described a 5 year radiographic overall survival of their revised hips of 91.5% [31]. The prevalence curves of painful and radiographically loose hips are very similar in shape as of revision year two (Figures 1 and 2) and we can at least carefully assume that the relationship between radiographic loosening and pain after revision THA is similar to that after primary THA.

### Weaknesses and strengths

The study has weaknesses and strengths that need to be considered. Only those patients were assessed who underwent follow-up examination before and after revision, whereas patients not attending follow-up visits may have changed the treatment center because of compromised outcomes or refused follow-ups due to satisfactory outcomes. Furthermore, the study represents a retrospective analysis of prospectively, systematically and consecutively collected data. Despite this setup, the multitude of centers included and the large time frame carry with them the potential for selection bias in such a non-monitored data collection endeavor. If there is selection bias, however, it should rather be a non-systematic one since none of the hospitals was aware of the goal of the current study and could have selectively included or excluded cases to their advantage. Therefore observed effects are rather diminished than amplified. In addition, only around 15% of patients had a record of 5 or more follow-ups. On the other hand, a large patient sample was assessed within a 10-year course around their first revision surgery and the core functional outcomes were reported based on current standards.

## Conclusion

Prevalence of radiological loosening signs and/or pain intensity follow an almost parallel course around the first revision of a THA for aseptic component loosening. This process begins about 4 years (radiographic loosening) before the actual revision surgery and intensifies about 2 years later (pain). It also involves walking capacity and hip range of motion. While pain levels go back to levels similar to those after primary surgery, range of motion and even more walking capacity remain moderately compromised.

## Abbreviations

BMI: Body mass index; IDES: International documentation and evaluation system; OP: Operation; RTHA: Revision total hip arthroplasty; THA: Total hip arthroplasty

## Competing interests

The authors declare that they have no competing interests.

## Authors’ contributions

EA is the principal investigator. He performed the study including statistical analysis and drafted the manuscript. RT and MN performed literature review and helped in drafting manuscript. EJL and MM helped in interpretation of the study results. SE and CR supervised the study and drafting the manuscript. CR conceived the study and participated in coordination and supervision of the study. All authors participated in the study design as well as read and approved the final manuscript.

## Pre-publication history

The pre-publication history for this paper can be accessed here:

http://www.biomedcentral.com/1471-2474/14/167/prepub
